# To: Adequacy of enteral nutritional support in intensive care units does not affect short- and long-term prognosis of mechanically ventilated patients: a pilot study

**DOI:** 10.5935/0103-507X.20200080

**Published:** 2020

**Authors:** Raquel Proença, André Gustavo Neves de Albuquerque, Geisy de Carvalho Alcantara, Valéria Lima da Cruz

**Affiliations:** 1 Department of Epidemiology, Instituto de Medicina Social, Universidade do Estado do Rio de Janeiro - Rio de Janeiro (RJ), Brazil.

**To the Editor**

In their original article “Adequacy of enteral nutrition support in intensive care units does not affect short- and long-term prognosis of mechanically ventilated patients: a pilot study”, Couto et. al. reported that critically ill patients provided ≥ 70% caloric intake during the first 72 hours following admission failed to show improved short- and long-term outcomes.^(1)^

It is informed in the title that it is a pilot study, however, a pilot study aims gathering data for planning and provide a rationale for larger research. Notwithstanding, the above-mentioned work appears to be a complete trial, as it aimed to assess a causal hypothesis: the relationship between exposure (nutritional intake ≥ 70%), and short- and long-term outcomes. Regarding the sample size, a pilot study may serve to make feasible a larger trial, providing data to estimate the losses and deaths magnitude and to help on the sample size calculation, among others.^(2)^

Therefore, considering that this is not a pilot study, we add below three substantial observations. First, in the discussion, the authors state that one of this study limitations was the lack of data collection regarding nutrients prescription and supply, but it is not clear how were the patients separated into the two different caloric supply groups (≥ 70% versus < 70%).

Second, even having the authors mentioned as limitations that no assessment of the patients’ functional capacity by the time of admission to the intensive care unit (ICU) was conducted, they conclude that the long-term functional capacity appears not to be influenced by the nutrition adequacy. We understand this conclusion not to be appropriate, as the patient could already have a low Lawton’s score before being admitted to the ICU.

Last, regarding the long-term outcomes, it is not possible to draw inferences, as the sample size was not large enough to allow this analysis. Therefore, presenting these results is not appropriate, and even less appropriate is drawing conclusions based on them. To calculate the number of patients for long-term analysis in a future study, we suggest using the data from this trial as a base to estimate how many patients are sufficient for this secondary analysis.

Some other general considerations also should be commented on. In table 2, the authors present data on short- and long-term outcomes plus mortality, however, each analysis has a different sample size, rendering reading the results unclear, as additionally, they do not agree with the table’s title. In this same table, results are presented only for the variable functional capacity, failing to mention details on how is this categorization made. Regarding the Lawton and Brody 1969 tool, the most appropriate reference would be Santos and Virtuoso Júnior,^(3)^ as the tool is already validated in Brazil. In the introduction, we missed references to support electing a 70% calory adequacy value as the cutoff point.
